# Increased Effectiveness of Microbiological Verification by Concentration-Dependent Neutralization of Sanitizers Used in Poultry Slaughter and Fabrication Allowing *Salmonella enterica* Survival

**DOI:** 10.3390/foods7030032

**Published:** 2018-03-03

**Authors:** Zahra H. Mohammad, Amer A. Hasan, Chris R. Kerth, David G. Riley, T. Matthew Taylor

**Affiliations:** 1 Department of Nutrition and Food Science, Texas A & M University, College Station, TX 77843, USA; zahra74@tamu.edu; 2 Department of Poultry Science, Texas A & M University, College Station, TX 77843, USA; dramir76@yahoo.com; 3 Department of Animal Science, Texas A & M AgriLife Research, College Station, TX 77843, USA; c-kerth@tamu.edu (C.R.K.); david-riley@tamu.edu (D.G.R.)

**Keywords:** sanitizer, neutralizer, *Salmonella*, peracetic acid, cetylpyridinium chloride, foodborne pathogens

## Abstract

Sanitizer neutralizers can assist foodborne pathogen detection during routine testing by counteracting sanitizer residues carried over into fluids collected and tested from food samples. This study tested sanitizer-matched neutralizers applied at increasing concentrations to facilitate *Salmonella enterica* survival following exposure to cetylpyridinium chloride (CPC) or peracetic acid (PAA), identifying minimum required concentrations of neutralizers to facilitate pathogen survival. *Salmonella* isolates were individually inoculated into a non-selective medium followed immediately by CPC (0.1 to 0.8% *v/v*) or PAA (0.0125 to 0.2% *v/v*) application, followed by neutralizers application. CPC was neutralized by lecithin and polysorbate 80, each supplemented into buffered peptone water (BPW) at 0.125 to 2.0X its respective content in Dey-Engley (D/E) neutralizing buffer. PAA was neutralized in BPW supplemented with disodium phosphate, potassium monophosphate, and sodium thiosulfate, each at 0.25 to 3.0X its respective concentration in BPW (phosphates) or D/E buffer (thiosulfate). Addition of neutralizers at 1X their respective concentrations in D/E buffer was required to allow *Salmonella* growth at the maximum CPC concentration (0.8%), while 2X neutralizer addition was required for *Salmonella* growth at the maximum PAA level (0.2%). Sanitizer neutralizers can assist pathogen survival and detection during routine food product testing.

## 1. Introduction

Currently in the United States of America, multiple food sanitizers are approved to decontaminate surfaces of poultry animal carcasses, as well as fabricated poultry parts (e.g., wings, thighs, breasts, drumsticks), from microbial pathogens such as *Salmonella enterica* and differing species of *Campylobacter* [1,2]. Nevertheless, sanitizer use may result in residues of the sanitizing agent being carried over into sample collection/rinsing fluid, potentially hindering subsequent pathogen detection [3]. The U.S. Department of Agriculture Food Safety and Inspection Service (USDA-FSIS) in July 2016 issued a notice to inspection personnel to initiate use of neutralizing buffered peptone water (nBPW) in lieu of the conventional BPW formulation for poultry carcass and parts verification sampling [4]. nBPW is formulated to contain three sanitizer neutralizing agents in addition to other BPW ingredients: sodium bicarbonate (NaHCO_3_; 12.5 g/L), sodium thiosulfate (Na_2_S_s_O_3_; 1.0 g/L), and refined soy lecithin (7.0 g/L) [5]. Lecithin and thiosulfate are also incorporated into the formula of Dey-Engley (D/E) neutralizing buffer; lecithin is reported to neutralize quaternary ammonium compounds (QACs) while thiosulfate can reduce chlorine and some other oxidizers [6,7]. Bicarbonate can assist the buffering of acidulants to prevent significant pH decline.

The USDA-FSIS currently enforces *Salmonella* performance standards for fresh poultry products and conducts routine sampling of inspected establishments in order to test compliance with the performance standard. At present, a maximum of 5/51 (9.8%) of poultry carcass samples, and 8/52 (15.4%) of poultry parts samples, are allowed to bear detectable *Salmonella* without the establishment falling out of compliance with the performance standard [8]. However, the impact of poultry sanitizer neutralizer use on *Salmonella* survival, as well as minimum neutralizer concentrations allowing pathogen survival following sanitizer application, remains unknown. This study was undertaken in order to determine the minimum neutralizer concentrations required to allow *Salmonella enterica* isolates comprising multiple serovars to survive exposure to two sanitizers approved for use in poultry harvest and fabrication: cetylpyridinium chloride (CPC) and peracetic acid (PAA). CPC is a quaternizing ammonium-type sanitizer allowed to a concentration of 0.8% by the U.S. Department of Agriculture in fresh poultry and poultry carcass sanitization, whereas PAA decomposes into acetic acid and hydrogen peroxide, and functions primarily as an oxidizing sanitizer and is allowed to a maximum concentration of 2000 ppm (0.2%) PAA [2].

## 2. Materials and Methods

### 2.1. Salmonella Revival and Inoculum Preparation

Three isolates of *S. enterica* subsp. *enterica* belonging to serovars Typhimurium, Kentucky, and Heidelberg were obtained from the Food Microbiology Laboratory (Department of Animal Science, Texas A & M University, College Station, TX, USA) culture collection; all isolates were recovered from a U.S. federally inspected poultry slaughter establishment. Isolates were revived from −80 °C by transfer of frozen cultures into tubes containing 9.0 mL sterile tryptic soy broth (TSB; Becton, Dickinson and Co., Franklin Lakes, NJ, USA) and incubating for 24 h at 35 °C. Following incubation, cultures were passed into sterile 9.0 mL TSB-containing tubes and incubated (24 h, 35 °C) prior to being prepared for inoculation. Inoculum fluid for each isolate was produced from revived cultures by serially diluting into 9.0 mL volumes of sterilized double strength TSB (2X TSB). Double strength TSB was used so as to provide the appropriate nutrient content to inoculated bacterial cells once sample preparation was completed. The targeted inoculum concentration for pathogen isolates in samples was 10^5^ CFU/mL. To quantify ingoing pathogen counts, decimal dilutions were prepared from 2X TSB tubes diluted to contain approximately 10^5^ CFU/mL in 0.1% (*w/v*) peptone diluent (Becton, Dickinson and Co.). *Salmonella* isolates were spread onto tryptic soy agar-containing (TSA; Becton, Dickinson and Co.) Petri plates; inoculated plates were incubated at 35 °C for 24 h prior to colony enumeration. Plate counts were log_10_-transformed for subsequent statistical analysis.

### 2.2. Neutralizer-Supplemented Buffered Peptone Water Preparation

BPW was selected as the neutralizer carrier fluid given its current usage in USDA-FSIS poultry sampling procedures [9]. Neutralizer-supplemented BPW was prepared from scratch using the formula published in the USDA-FSIS Microbiology Laboratory Guidebook [10] to allow alteration of the formulation as needed. To prepare neutralizer-supplemented BPW matched for CPC, 40 mL BPW was added to a 100-mL volumetric flask. Stock solutions of refined soy lecithin and polysorbate 80 (Sigma-Aldrich Co., St. Louis, MO, USA) were prepared in distilled water so as to obtain two times (2X) their standard concentrations in D/E buffer (soy lecithin: 7.0 g/L; polysorbate 80: 5.0 g/L) upon completion of final sample preparation. After addition of lecithin and polysorbate 80, sufficient BPW was added to bring to volume (100 mL). Similar preparations of CPC neutralizers-supplemented BPW were prepared to provide 1.0X, 0.5X, 0.25X, and 0.125X the standard contents of neutralizers in D/E buffer in like fashion. All solutions were then transferred into screw-cap bottles, subjected to steam sterilization (15 min, 121 °C), and tempered to room temperature prior to use. Peracetic acid neutralizers-supplemented BPW was prepared in similar fashion to BPW prepared for CPC experiments. Neutralizers for PAA were sodium thiosulfate, disodium phosphate (Na_2_HPO_4_), and monopotassium phosphate (KPO_4_; Sigma-Aldrich Co.). For thiosulfate and phosphates, BPW basal medium (1% peptone, 0.5% NaCl, distilled water; not containing phosphates) was supplemented with sufficient neutralizers to provide up to three times (3X) each of neutralizers’ respective contents in BPW or D/E buffer upon final sample preparation (thiosulfate: 6.0 g/L; disodium phosphate: 3.5 g/L; monopotassium phosphate: 1.5 g/L) [10]. Similar preparations were then produced to deliver 2X, 1X, 0.5X, and 0.25X of neutralizers’ respective concentrations in individual flasks, steam-sterilized (15 min, 121 °C), and then cooled to ambient before use.

### 2.3. Sanitizer Preparation

Commercial CPC (Cecure^®^, 40% CPC; Safe Foods Corp., North Little Rock, AR, USA) was diluted in sterile distilled water in a 100-mL volumetric flask. CPC was added to deliver a final concentration of 0.8% CPC upon final sample preparation (1.0 mL 2X TSB inoculated with *Salmonella* isolate +0.5 mL sanitizer +0.5 mL CPC-matched neutralizers-supplemented BPW). Similar working stocks, designed to deliver 0.6, 0.4, 0.2, and 0.1% (*v/v*) CPC, were prepared in like fashion for experimental use. In the case of PAA, working stocks of commercial PAA solution (Promoat^TM^, Safe Foods Corp., Rogers, AR, USA) were prepared in similar fashion as those for CPC experiments, and were used within 30 min of preparation based on manufacturer guidance regarding PAA degradation kinetics upon dilution. PAA working stocks were prepared to deliver 0.2, 0.1, 0.05, 0.025, and 0.0125% (*v/v*) PAA upon completion of sample preparation. For both sanitizer solutions, upon mixing of working stocks, sanitizer solutions were filtered through a 0.45 µm filter.

### 2.4. Experimental Procedures

To initiate sanitizer neutralization experiments, 1.0 mL volumes of *Salmonella* isolates diluted in 2x TSB were aseptically loaded into sterilized, cooled (25 °C) glass vials. Then, 0.5 mL of sanitizer solution was added aseptically. Finally, 0.5 mL of appropriately mixed sanitizer (CPC or PAA)-matched neutralizer-supplemented BPW was added into the sample vial. Procedures were completed to yield all combinations of five sanitizer concentrations and five neutralizer additions for each *Salmonella* isolate. Additionally, sanitizer-containing, neutralizer-free controls were prepared by adding 1.0 mL pathogen-inoculated 2X TSB, 0.5 mL sanitizer, and 0.5 mL sterile distilled water to confirm sanitizer inactivation of pathogens absent of neutralizer addition. Once sample preparations were completed, samples were capped and incubated for 24 h at 35 °C. Following incubation, tubes were checked for turbidity as evidence of pathogen survival and growth, and scored for *Salmonella* isolate growth (1) or no growth (0). Sample tubes not showing visible evidence of growth were sampled for *Salmonella* survival by spreading a 0.1 mL volume onto the surface of a TSA Petri dish. Following inoculation, plates were incubated for 24–36 h at 35 °C prior to checking for colonies. If colonies were detected following incubation, the source tube was scored as positive (1) for *Salmonella* survival.

### 2.5. Analysis of Data

Sanitizer neutralization experiments were replicated six times in identical fashion (*N =* 6). *Salmonella* isolate-specific mean inoculum (log_10_ CFU/mL) were first compared to one another by Student’s *t*-test (*p* < 0.05) in order to determine whether inoculum counts between isolates differed. Sets of ‘1’s and ‘0’s (growth and no growth, respectively) for each *Salmonella* isolate and sanitizer/neutralizer combination were analyzed by two-way analysis of co-variance (ANCOVA). Log_10_-transformed counts of *Salmonella* isolate inocula were treated as a co-variate to detect and negate any potential influence on growth/no growth results as a function of *Salmonella* inoculum levels. Least squares means of *Salmonella* growth/no growth results were subsequently separated by Student’s *t*-test, assigning significance at *p* = 0.05. Chi square (χ^2^) analysis was completed to determine whether *Salmonella* growth/no growth frequencies were independent of sanitizer and neutralizer application (H_0_) or dependent upon sanitizer and neutralizer application (H_1_), at a significance level of *p =* 0.05. Finally, a logistic regression model was fitted identifying the response variable *Salmonella* survival (1 = growth; 0 = no growth) as a binomially distributed random variable. Growth was evaluated using generalized linear mixed models, with distinct models for each sanitizer [11]. Logit link functions were employed during analysis; the residual variance was fixed at a value of 1. The replicate was modeled as a random effect. Multiple parameterizations of sanitizer levels and neutralizer levels were initially investigated. The parameterization that appeared most appropriate for characterizing *Salmonella* response to sanitizer and neutralizer application was the ratio of neutralizer concentration to sanitizer concentration as a continuous variable. Then, linear, quadratic, and cubic regression coefficients for those ratios as covariates were investigated to determine the optimal model for characterizing growth/no growth responses of *Salmonella* for each sanitizer.

## 3. Results

### 3.1. Survival of Salmonella Isolates as a Function of CPC and CPC-Matched Neutralizer Combinations

The application of sanitizer-matched neutralizers in BPW was anticipated to counteract antimicrobial activity of CPC and PAA sanitizers at sanitizer-specific minimum required neutralizer concentrations. Mean counts of *Salmonella* Typhimurium, Kentucky, and Heidelberg inocula for CPC experiments were 5.48 ± 0.31, 5.58 ± 0.42, and 5.90 ± 0.11 log_10_ CFU/mL, respectively. *Salmonella* isolate mean inoculum counts did not differ from one another (*p* > 0.05). For *Salmonella* challenged with CPC and then neutralized, the interaction of sanitizer concentration and neutralizers concentration was highly significant (*p* < 0.01) for predicting *Salmonella* growth or non-growth; counts of *Salmonella* inocula did not function as a co-variate to influence growth/no growth results (Table 1). Addition of neutralizers at 1X their respective contents in D/E buffer was required to allow for *Salmonella* isolates’ survival exposed to 0.6 and 0.8% CPC, except during one replicate testing *S.* Typhimurium. Predictably, as CPC content was lowered, required contents of sanitizer neutralizers allowing *Salmonella* survival were reduced, except that even 0.1% CPC (lowest applied sanitizer concentration) was generally capable of inhibiting *Salmonella* growth when challenged with 0.125X neutralizers addition (Table 1). A *Χ*^2^ = 408.5 (*p* < 0.01) was obtained for *Salmonella* isolates treated with CPC and subsequently neutralized, indicating *Salmonella* growth or non-growth was dependent upon sanitizer and neutralizers combinations, rejecting the null hypothesis. No *Salmonella* growth was detected in control samples where isolates were challenged with the lowest concentration of sanitizer but not neutralized.

### 3.2. Survival of Salmonella as a Function of PAA and PAA Neutralizer Combination

Mean *Salmonella* inoculum counts for PAA experiments were 5.63 ± 0.43, 5.35 ± 0.62, and 5.64 ± 0.29 log_10_ CFU/mL for *S.* Typhimurium, Kentucky, and Heidelberg, respectively. Mean inoculum counts did not differ statistically between *Salmonella* isolates (*p* > 0.05). Similar to CPC results (Section 3.1), *Salmonella* isolates did not differ in responses (growth, non-growth) to sanitizer and matched neutralizers application (*p* > 0.05) (Table 2). *Salmonella* inoculum counts did not function as a co-variate to influence pathogen growth for sanitizer and neutralizers pairings (Table 2). Compared to CPC, 2X neutralizers addition was required to support *Salmonella* growth at the highest applied PAA concentration (0.2% *v/v*; Table 2). At intermediate concentrations of PAA (0.05, 0.1%), statistical differences were observed with respect to frequency of *Salmonella* survival as a function of neutralizer content (*p* < 0.05), but not as a function of *Salmonella* isolate. For 0.05% PAA sanitizer, increasing from 0.5 to 1.0X neutralizers addition resulted in a statistically significant increase in *Salmonella* survival frequency (Table 2). Similar to results for *Salmonella* treated with CPC and neutralizers (Section 3.1), *Salmonella* growth was dependent upon PAA + neutralizers application (*χ*^2^ = 369.4; *p* < 0.01). Consistent growth for all isolates was observed at the lowest concentrations of PAA (0.0125, 0.025%) except at the lowest neutralizers addition (0.5X, 0.25X).

### 3.3. Modeling of Salmonella Survival As a Function of Sanitizer and Combined Neutralizers Exposure via Logistic Regression

**Figure 1:**
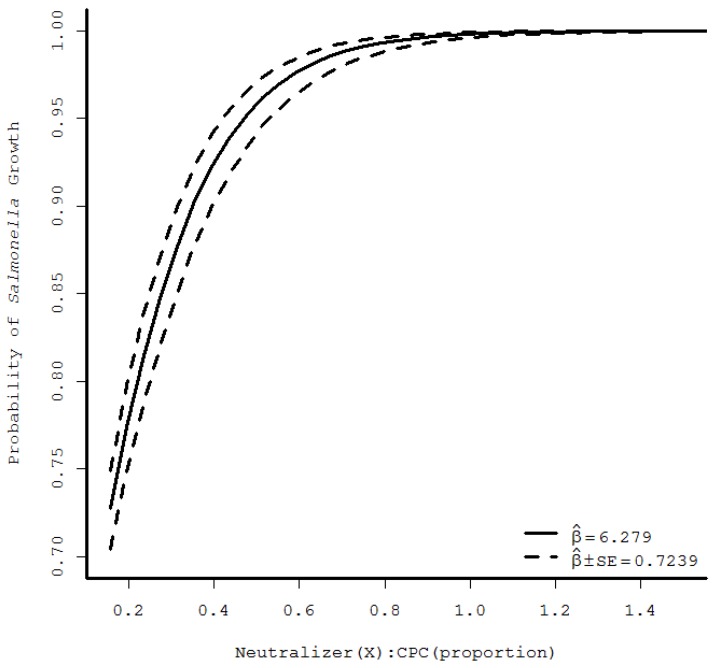
Probability of *Salmonella* growth following exposure to cetylpyridinium chloride (CPC) and matched neutralizers (lecithin, polysorbate 80), depicting predicted values and confidence band representing one standard error from the linear regression of logit values versus the ratio of neutralizers:sanitizer (*N* = 6). SE: standard error of model.

**Figure 2:**
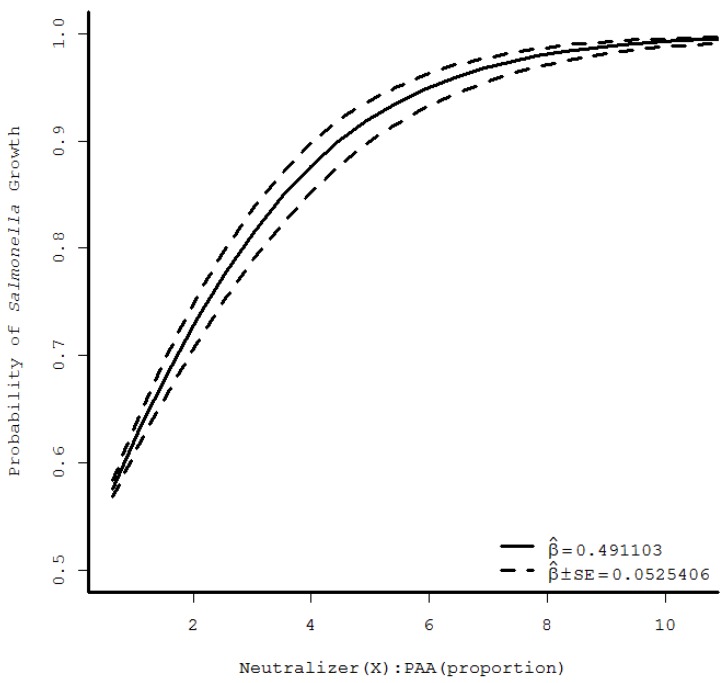
Probability of *Salmonella* growth following exposure to peracetic acid (PAA) and matched neutralizers (sodium thiosulfate, disodium phosphate, potassium phosphate), depicting predicted values and confidence band representing one standard error from the linear regression of logit values versus the ratio of neutralizers:sanitizer (*N* = 6). SE: standard error of the model.

During logistic regression analysis, increasing the order of the regression from linear to quadratic to cubic resulted in highly significant *F*-statistics for each analysis unique to CPC and PAA-specific datasets. Plots of the predicted curves were evaluated on logit and also on observed scales after application of the inverse link function. Throughout the various models’ analysis, linear regression appeared to most appropriately characterize *Salmonella* growth across the range of experimental neutralizers/sanitizer combinations. Plots of the predicted *Salmonella* growth/no growth as a function of the ratio of neutralizers:sanitizer probability are shown in Figure 1 and Figure 2 for CPC and PAA, respectively. From these, minimum ratios of neutralizers:sanitizer of 0.68 and 8.8 for CPC and PAA, respectively, were identified to provide high likelihood of *Salmonella* growth (probability of growth > 0.99). Figure 1 and Figure 2 demonstrate convergence of observed *Salmonella* growth/no growth response means with standard error limits at the lower and higher ratios of neutralizers/sanitizer for CPC and PAA, respectively.

## 4. Discussion

Research testing the survival of human pathogens following sanitizer exposure has indicated *Salmonella* may be inhibited by sanitizers such as CPC and PAA during sanitizer testing during poultry slaughter and/or meat fabrication. Kim and Slavik [12] reported 0.1% CPC spray or immersion application reduced *S. Typhimurium* on chicken skin by 0.9 to 1.8 log_10_ CFU/cm^2^, suggesting the sanitizer permeabilized the outer membrane of the pathogen as well as inhibited proper metabolism. Others have shown significant reductions in *Salmonella* counts on chicken carcasses following application of 0.02 to 0.04% PAA [13,14]. Nevertheless, Gamble et al. [3] reported the capacity of *Salmonella* to survive CPC (0.8%) or PAA (0.2%) supplementation in BPW under conditions designed to simulate extended poultry carcass dripping (5 min) occurring during routine verification sampling. Researchers concluded neutralizers usage in poultry sampling fluids was warranted to reduce the potential of pathogen non-detection. Soon thereafter, USDA-FSIS released Notice 41-16 directing the implementation of nBPW use during routine testing to enhance the likelihood of pathogen detection during poultry routine verification testing [4].

In the current study, data analysis indicates *Salmonella* detection would be supported when CPC-matched neutralizers are added into poultry rinsing fluids at 1X their respective concentrations in D/E neutralizing buffer. The formula for nBPW contains refined soy lecithin at 7.0 g/L and sodium thiosulfate at 1.0 g/L [5]. Assuming a poultry slaughter facility applies CPC at its maximum allowable concentration of 0.8% to poultry surfaces, even without loss of CPC activity prior to sample collection, *Salmonella* detection would be facilitated by nBPW as its CPC-matched neutralizers contents are equivalent to that of D/E neutralizing buffer (Table 1) [10]. Furthermore, the required ratio of CPC neutralizers:CPC of 0.68 indicates only moderate amounts of neutralizers are required for sanitizer neutralization under conditions likely optimal for contaminating pathogens (no sub-lethal injury onset by previously applied interventions, no impacts of cold water application typical of immersion chilling) (Figure 1).

In contrast to CPC findings, *Salmonella* detection following application of PAA as impacted by PAA-matched sanitizers indicated a higher neutralizers requirement for predictable pathogen detection. While frequencies of pathogen detection as a function of sanitizer and neutralizers pairings did not differ between CPC and PAA, the requirement of at least 2X the standard concentrations of neutralizers in BPW or D/E media for PAA neutralization indicates greater opportunity for pathogens to escape detection on poultry samples if the sanitizer is insufficiently neutralized. The predicted requirement for 8.8 times the neutralizer content versus sanitizer addition indicates greater neutralizer addition requirement for pathogen detection. This may have occurred due to decreased acidulant buffer content in the 1X experimental PAA neutralizer fluid (3.5 g/L sodium phosphate dibasic +1.5 g/L potassium phosphate monobasic) [10] vs. nBPW formulation (12.5 g/L sodium bicarbonate) used in USDA-FSIS routine testing [5]. In the current study, the combination of phosphates, as compared to bicarbonate, was used in order to provide for acidulant neutralization/buffering. This was done due to the inclusion of these agents in BPW as well as due to a lack of awareness of USDA-FSIS’s intent for formulation of nBPW at the time of experiment startup. Repeat of the current study method, substituting bicarbonate for phosphates, may lead to differing outcomes regarding minimal requirements for PAA neutralizers use.

The multiple forms of analysis applied in the current study provided a more comprehensive understanding of the data and experimental outcomes. The confirmation that *Salmonella* inoculum counts (log_10_ CFU/mL) did not differ from one another, and that *Salmonella* inoculum did not function as a covariate to influence pathogen growth/no growth, demonstrated *Salmonella* serovars are not likely to differ in their response to an applied sanitizer. ANCOVA and *Χ*^2^ analyses both indicated that *Salmonella* growth was influenced by the content of sanitizer and neutralizer applied, though these procedures do so by differing means. Finally, the utilization of logistic regression provided opportunity to predict *Salmonella* growth/no growth along a continuum of predicted combinations of sanitizer and matched neutralizers, not available via analysis of variance or *Χ*^2^ procedures. This technique facilitates prediction of minimum needs of neutralizers for refining nBPW formulation, or for application of neutralizers into testing media in other applications. Previous research has indicated the superiority of logistic regression of binary data versus linear regression [15]. In the current study, whereas the ratios of sanitizer to neutralizers numerically differed for CPC and PAA, frequencies of *Salmonella* growth/no-growth did not differ for the two sanitizers, supporting the utility of logistic regression analysis in tandem with ANOVA for gaining more in-depth understanding of potential pathogen survival outcomes during food sanitizer use and subsequent pathogen testing. Ultimately, the utilization of multiple forms of data analysis yielded a comprehensive understanding of the impact of sanitizer neutralization during experimental trials and provided multiple points of reference for subsequent study of *Salmonella* recovery as impacted by sanitizer and neutralizers application.

## 5. Conclusions

In summary, data presented herein indicate that non-injured cells belonging to *Salmonella enterica* serovars do not differ in their responses to the USDA-FSIS-approved poultry sanitizers CPC and PAA applied in vitro. While *Salmonella* inocula did not influence outcomes of growth/no growth experiments, *Salmonella* growth or no growth was dependent upon, and highly influenced by, the contents of paired sanitizer and matched neutralizers (*p* < 0.01). Predicted neutralizer requirements for consistent sanitizer neutralization were determined through multiple methods of data analysis. The current study tested neutralizers and sanitizers mixed in liquid medium that likely allowed for homogeneous mixing of compounds and bacterial cells in the sample vial. Sanitizer neutralizers, when matched to the sanitizer and of sufficient concentration, can facilitate pathogen survival and subsequent detection during sampling of a food product.

## Figures and Tables

**Table 1:** Least squares means of the proportions of *Salmonella* growth or non-growth as affected by the interaction of cetylpyridinium chloride (CPC) concentration and combined neutralizers addition (*p* < 0.01).

Neutralizer Addition ^1^	0.8% ^2^	0.6%	0.4%	0.2%	0.1%
2X	1.0 A ^3^	1.0 A	1.0 A	1.0 A	1.0 A
1X	1.0 A	1.0 A	1.0 A	1.0 A	1.0 A
0.5X	0.0 D	0.06 D	0.94 A	1.0 A	1.0 A
0.25X	0.0 D	0.0 D	0.0 D	0.5 B	1.0 A
0.125X	0.0 D	0.0 D	0.0 D	0.0 D	0.28 C
Pooled Standard Error = 0.04					

^1^ 1X concentrations for sanitizer (CPC; Cecure^®^, 40% CPC, Safe Foods Corp., North Little Rock, AR, USA) neutralizers were 7.0 g/L lecithin and 5.0 g/L polysorbate 80 [10]; ^2^ CPC was formulated in distilled water to concentrations sufficient to produce indicated final concentrations upon completion of all additions to sample vials (1.0 mL diluted *Salmonella* culture in 2X TSB +0.5 mL CPC stock +0.5 mL neutralizers-supplemented BPW); ^3^ Values presented are compiled from six independently completed replicates, with one sample per replicate (*N* = 6). Values not sharing common letters (A–D) differ by two-way analysis of variance and Student’s *t*-test means separation test at *p* = 0.05. *Salmonella* isolate inoculum counts (log_10_ CFU/mL) did not function as a co-variate to impact *Salmonella* growth/no growth results (*p* > 0.05).

**Table 2:** Least squares means of the frequencies of *Salmonella* growth or non-growth (number of samples showing growth or non-growth/total number samples) as affected by the interaction of peracetic acid (PAA) concentration x combined neutralizers addition (*p* < 0.0001).

Neutralizer Addition ^1^	0.2% ^2^	0.1%	0.05%	0.025%	0.0125%
3X	1.0 A ^3^	1.0 A	1.0 A	1.0 A	1.0 A
2X	1.0 A	1.0 A	1.0 A	1.0 A	1.0 A
1X	0.0 D	0.06 D	0.94 A	1.0 A	1.0 A
0.5X	0.0 D	0.0 D	0.0 D	0.5 B	1.0 A
0.25X	0.0 D	0.0 D	0.0 D	0.0 D	0.28 C
Pooled Standard Error = 0.05					

^1^ 1X concentrations for sanitizer (PAA; Promoat™, 11–13% PAA, Safe Foods Corp., North Little Rock, AR, USA) neutralizers were Na_2_S_2_O_3_ (6.0 g/L), Na_2_HPO_4_ (3.5 g/L) and KPO_4_ (1.5 g/L) [10]; ^2^ PAA was formulated in distilled water to concentrations sufficient to produce indicated final concentrations upon completion of all additions to sample vials (1.0 mL diluted *Salmonella* culture in 2X TSB +0.5 mL PAA stock +0.5 mL neutralizers-supplemented BPW); ^3^ Values presented are compiled from six independently completed replicates, with one sample per replicate (*N* = 6). Values not sharing common letters (A–E) differ by two-way analysis of variance and Student’s *t*-test means separation test at *p* = 0.05. *Salmonella* isolate inoculum counts (log_10_ CFU/mL) did not function as a co-variate to explain *Salmonella* growth/no growth results (*p* > 0.05).
